# A Bi-fluorescence complementation system to detect associations between the Endoplasmic reticulum and mitochondria

**DOI:** 10.1038/s41598-017-17278-1

**Published:** 2017-12-12

**Authors:** Mark Harmon, Philip Larkman, Giles Hardingham, Mandy Jackson, Paul Skehel

**Affiliations:** 1 Centre for Integrative Physiology, Euan MacDonald Centre for Motor Neurone Disease Research, The University of Edinburgh, Edinburgh EH8 9XD, UK; 2 UK Dementia Research Institute at the University of Edinburgh, Edinburgh Medical School, Edinburgh EH8 9XD, UK

## Abstract

Close contacts between the endoplasmic reticulum membrane and the mitochondrial outer membrane facilitate efficient transfer of lipids between the organelles and coordinate Ca^2+^ signalling and stress responses. Changes to this coupling is associated with a number of metabolic disorders and neurodegenerative diseases including Alzheimer’s, Parkinson’s and motor neuron disease. The distance between the two membranes at regions of close apposition is below the resolution of conventional light microscopy, which makes analysis of these interactions challenging. Here we describe a new bifluorescence complementation (BiFC) method that labels a subset of ER-mitochondrial associations in fixed and living cells. The total number of ER-mitochondria associations detected by this approach increases in response to tunicamycin-induced ER stress, serum deprivation or reduced levels of mitofusin 2 (MFN2). This method will facilitate the analysis of dynamic interactions between the ER and mitochondrial membranes.

## Introduction

Regions of close association between the outer membrane of mitochondria (OMM) and the endoplasmic reticulum (ER) were originally identified by ultrastructure analysis^1,2^, and subsequently shown to represent specialised structures that facilitate coordinated Ca^2+^ transfer and signalling between the two organelles^3^. These regions of the ER membrane have been biochemically purified as Mitochondrial Associated Membranes, or “MAMs”, and shown to be enriched for a range of proteins including enzymes responsible for lipid synthesis^4^ and transfer between the two membrane systems^5–7^. mtDNA synthesis and mitochondrial division appear to be co-ordinated at sites of close apposition with the ER^8,9^, as is the formation of the autophagosome^10^. More recently the properties of ER-mitochondrial junctions have been implicated in a number of pathologies. Metabolic activities associated with MAMs are increased in Alzheimer’s Disease^11,12^ and transgenic mice expressing P301L tau appear to have more ER membrane associated with mitochondria^13^. In familial Parkinson’s loss of DJ-1 reduces ER-mitochondria associations and disrupts mitochondrial Ca^2+^ homeostasis^14^ and α-synuclein has been localized to MAMs^15,16^. An interaction between the ER protein VAPB and mitochondrial PTPIP51/Rmdn3 influences the coupling of Ca^2+^ levels in these organelles by a mechanism that is sensitive to TDP-43 activity^17^ thus linking ER-mitochondrial interactions to both spontaneous and familial forms of Amyotrophic Lateral Sclerosis^18–20^. In addition the metabolic homeostasis signalling through ER-mitochondrial contacts is suggested to have a central role in metabolic diseases^21,22^.

Close appositions between the ER and OMM measured in fixed cells using electron microscopy vary in distance between 5–30 nm and the extent of mitochondrial area covered by the ER depends on cell type and metabolic activity^3,23^. Membrane associations have been detected and quantified by co-localisation analysis of fluorescent signals targeted to the separate membranes^24^, but this has a limited spatial resolution of 150–200 nm and cannot, therefore, distinguish between ER/OMM contacts of differing distance. Alternative methods to detect and quantify ER mitochondrial associations have been successfully developed based on proximity ligation in fixed cells^25^, luciferase complementation and dimerization-dependent fluorescent protein (ddFP) approaches in living cells^26,27^. Here we report a Bimolecular Fluorescence Complementation BiFC system^28^ in which two complementing fragments of a fluorescent protein are expressed on the ER or mitochondria. When the membranes of these organelles are in very close apposition the two complementing fragments of the fluorescent protein can associate and reconstitute a functional fluorescent protein. The BiFC signal is only generated when the OMM and the cytoplasmic face of the ER are in close apposition. Therefore, this method provides structural information at a higher resolution than co-localisation and with a lower background than ddFP. As with ddFP this method may readily be applied to follow the dynamics and distribution of membrane juxtapositions within living cells in real time. Furthermore, as the complementing fragments can only produce fluorescence when directly interacting and are not fluorescent individually, the quantification of organelle contacts with BiFC is potentially less sensitive to changes in the local protein concentrations protein concentrations than ddFP methods.

## Results

**Figure 1:**
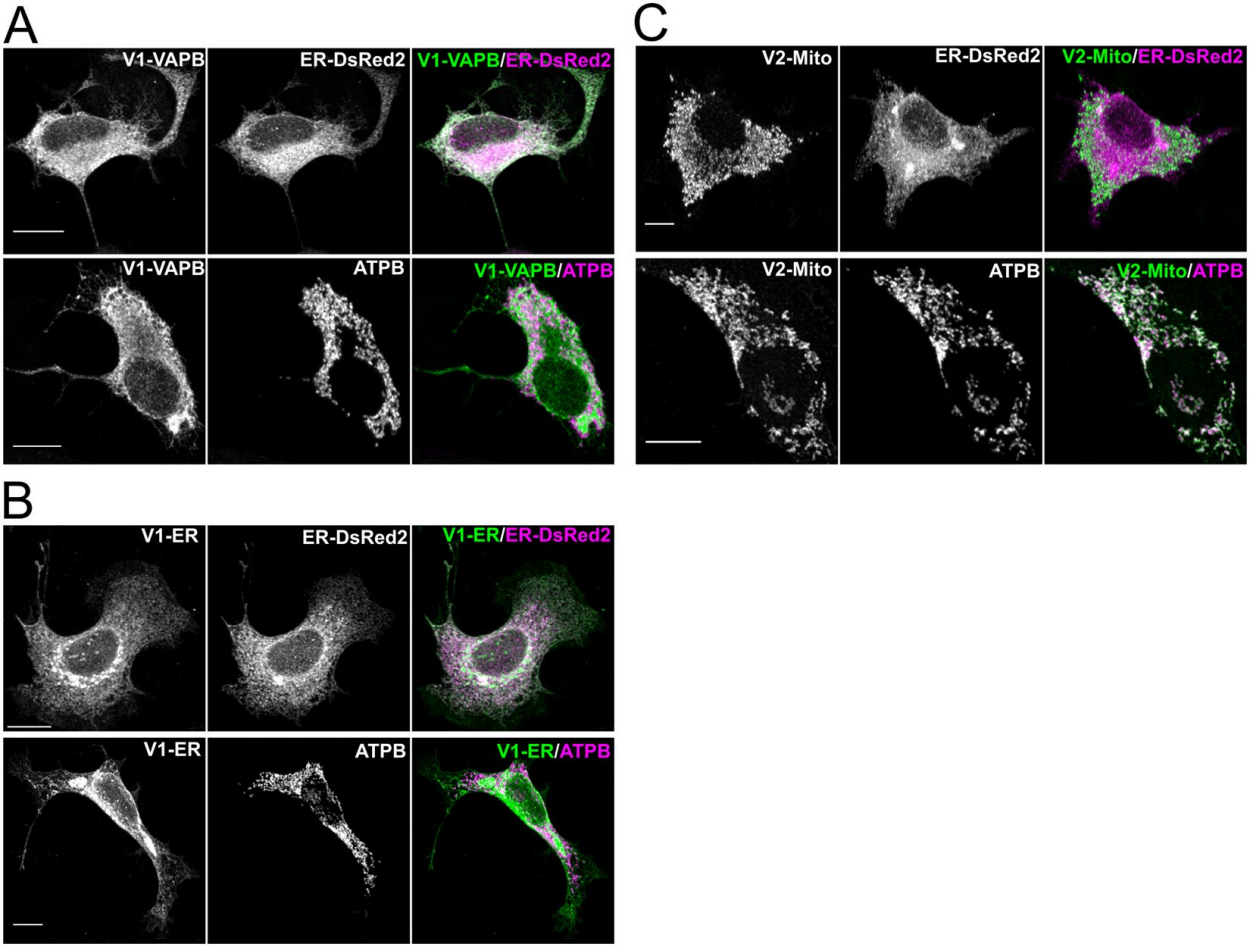
The complementing YFP Venus fusion proteins are correctly targeted to the ER and mitochondria. NSC 34 cells transiently expressing V1-VAPB (**A**), V1-ER (**B**), or V2-Mito (**C**), were analysed by immunocytochemistry using an anti-GFP antibody that recognises both fragments of YFP. The endoplasmic reticulum was identifed by co-expression of ER-DsRed2 (Clontech), and mitochondria were labelled immunolcytochemically for ATPB. The extent of colocalisation of Venus fusion proteins with the orgamelle markers was analysed by Pearon’s Correlation Coefficient (PCC) (Table 1). Values indicate mean PCC ± SEM; n = 3 independent experiments (9–10 cells total). Antibody details Table 3. Scale bars, 10 μm.

**Figure 2:**
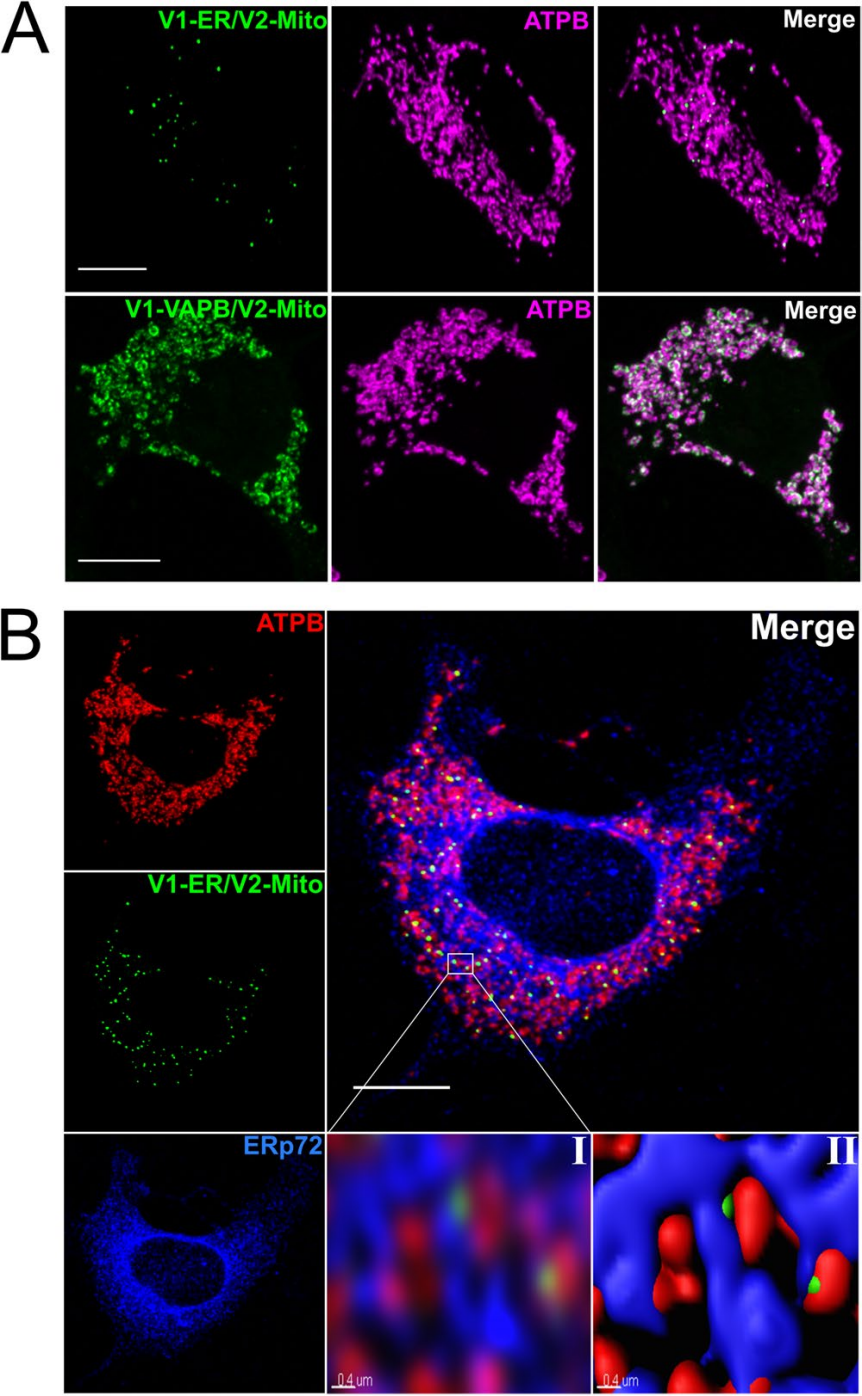
ER-targetted Venus 1 fusion proteins generate BiFC signals when co-expressed with mitochonrial Venus 2. V1-ER and V1-VAPB were co-expressed with V2-Mito in NSC34 cells (**A**). The resulting BiFC signal was associated with mitochondria as indicated by ATPB expression. The two Venus 1 fusion proteins differ in length by 218 aa, and produce distinct subcellular BiFC paterns. Triple labelling of the ER (Erp72, *blue*) and mitochondria (ATPB, *red*) demonstrate that the V1-ER/V2-Mito BiFC lables descete puncta at junctions between the ER and mitochodria (**B**). Higher magnification of boxed region (B.I), processed and rendered in 3D by Imaris image analysis software (**B.II**). Scale bar 10 μm, or 0.4 μm in panels B.I and B.II.

To generate a BiFC reporter for the close apposition of the ER and OMM the two complementing fragments of the split YFP Venus protein were targeted separately to the ER and OMM. In principle a functional YFP will only be generated at regions where the two membranes are sufficiently close for the direct interaction of the two complementing Venus fragments. The N-terminal fragment Venus1^28^ was targeted to the cytoplasmic face of the ER membrane by fusion to either the full length or the C-terminal 25 amino acids of the mouse VAPB protein, creating V1-VAPB and V1-ER respectively. The C-terminal fragment Venus 2 was targeted to the outer mitochondrial membrane by fusion to the first 34 amino acids of TOMM20, to generate V2-Mito^29^. Limiting the size of the targeting sequences ensures that only the closest associations of the membranes will be detected. The sub-cellular localisation of these fusion proteins in the cell line NSC34 was determined by immunocytochemical analysis using a polyclonal anti-serum against GFP that recognises both N- and C-terminal fragments of the Venus protein (Fig. 1). The immunofluorescent signal from V1-VAPB co-localized with the co-expressed ER marker ER-DsRed2 (Clontech), but not with the mitochondrial protein ATPB (Fig. 1A and Table 1). Similarly, V1-ER also co-localized with the ER marker, but not with the mitochondrial protein ATPB (Fig. 1B and Table 1). In contrast, V2-Mito co-localized with ATPB but not with co-expressed ER-DsRed2 (Fig. 1C, Table 1). Co-expression of either V1-VAPB or V1-ER with V2-Mito in NSC34 cells produced readily detectable BiFC signals (Fig. 2). V1-VAPB/V2-Mito produced a fluorescence pattern surrounding almost all mitochondria, similar to that reported by dimerization-dependent fluorescent proteins targeted to the ER and OMM^27^. Co-expression of the shorter V1-fusion protein, V1-ER with V2-Mito generated a distinct fluorescent signal with discrete puncta of fluorescence associated with a subset of mitochondria (Fig. 2). These signals appear to lie exclusively at areas of close apposition of the ER and mitochondria as revealed by immunolabelling for ERp72 and ATPB respectively (Fig. 2). Expression of either V1-VAPB, V1-ER or V2-Mito individually produced no YFP signal (Fig. S1). Co-expression of V1-ER with V2-Mito over 48 hrs produced no gross changes to ER or mitochondria morphology in NSC34, COS7 or HEK293 cells (Fig. S2). The V1-ER with V2-Mito BiFC was further characterised in the remainder of the study.

**Table 1:** Fluorescent signal co-localisation quantified by Pearson’s Correlation Coefficient (PCC).

V1-VAPB/ER-DsRed2	0.876 ± 0.009
V1-VAPB/ATPB	0.035 ± 0.010
V1-ER/ER-DsRed2	0.870 ± 0.017
V1-ER/ATPB	0.070 ± 0.020
V2-Mito/ER-DsRed2	0.050 ± 0.011
V2-Mito/ATPB	0.910 ± 0.008

It would be anticipated that if the presence of V1-ER and V2-Mito was inducing ER-mitochondria associations as an artefact, then increasing the expression levels of V1-ER and V2-Mito would distort ER morphology and lead to an increase in the number or size of BiFC signals, but this was not the case (Fig. S3). Transfection with increasing amounts of V1-ER and V2-Mito expression plasmid DNA lead to an increase in the number of fluorescent cells but the average number of fluorescent signals in any transfected cell was unaltered (Fig. S3). Therefore, the presence of V1-ER and V2-Mito seems not to directly induce the formation of membrane associations, but labels existing sites of close apposition between the ER and OMMs.

**Figure 3:**
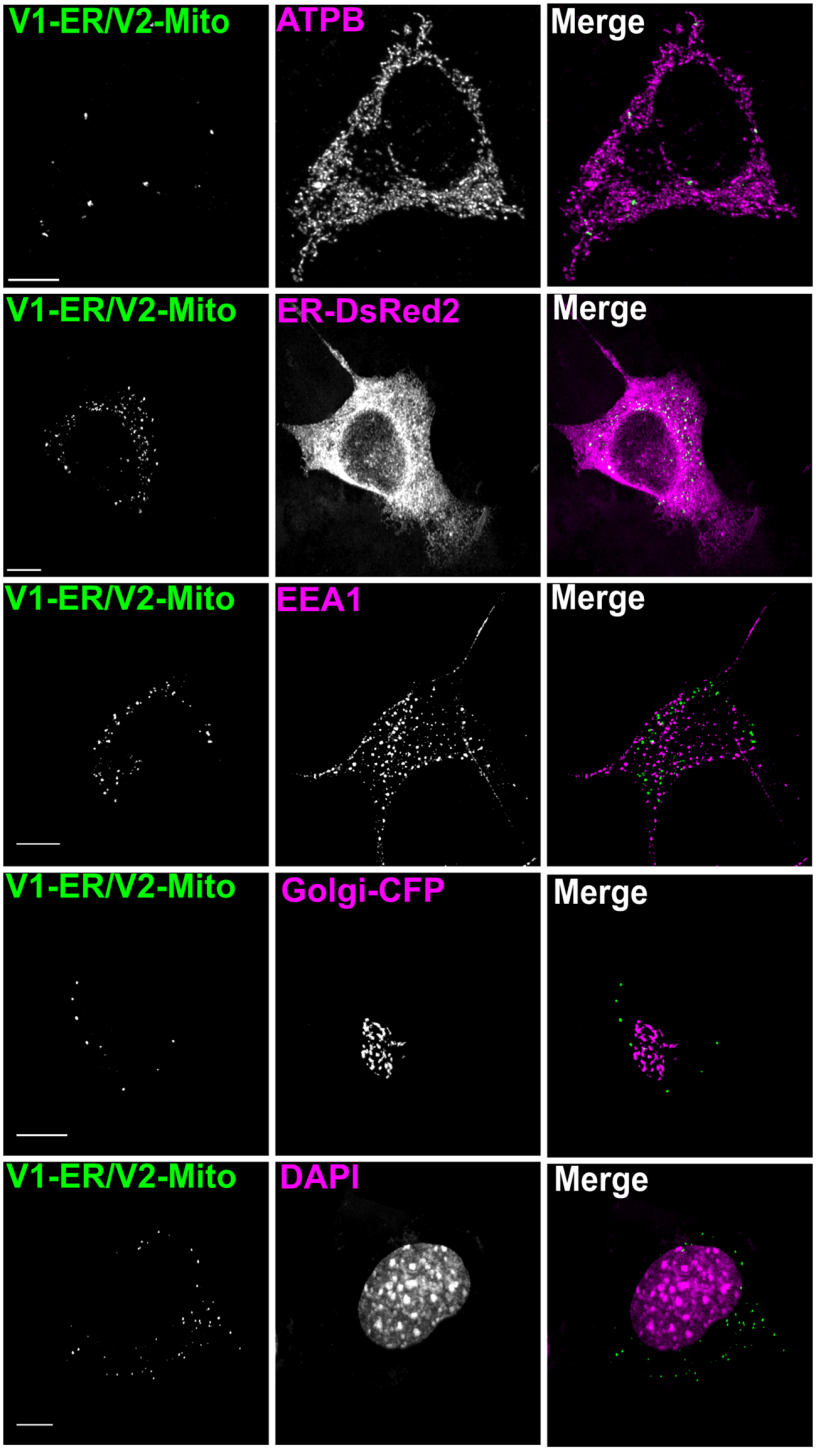
Quantification of V1-ER/V2-Mito BiFC signal overlapping with organelle markers. The number of BiFC puncta in NSC 34 cells expressing V1-ER/V2-Mito was quantified using a maximum intensity threshold in Imaris image analysis software **(A)**. The co-localisation channels were then built between the YFP channel and fluorescent organelle markers. The ER and Golgi were labeled by co-expression of ER-DsRed2 and Golgi-ECFP respectively (Clontech), early endosomes and mitochondria were immunolabeled for EEA1 or ATPB, and the nucleus was labeled with DAPI. anti-ATPB (mitochondria), ER-DsRed (ER), anti-EEA1 (Endosomes), Golgi-CFP (Golgi), DAPI (Nucleus). Using the same intensity threshold value, the number of BiFC puncta that had a point of colocalisation with the fluorescent organelle signal were quantified. n = 3 independent experiments (10–15 cells total). Scale bars, 10 μm. Values indicate the mean number of colocalised BiFC puncta over three independent experiments ± SEM (Table 2, Antibody details Table 3).

The ER comes in close apposition to other organelles including the Golgi apparatus and endosomes^30–33^. However the BiFC puncta generated by V1-ER and V2-Mito appear selective for the ER and OMM, with very few signals associated with the Golgi apparatus, the nucleus or EEA1-positive endosomes (Fig. 3, Table 2). Details of the antibodies used are shown in Table 3.

**Table 2:** % of BiFC puncta overlapping with fluorescent organelle marker.

ATPB (mitochondria)	97.07 ± 1.34
ER-DsRed2 (ER)	99.07 ± 0.27
EEA1 (Endosomes)	10.74 ± 2.36
Golgi-CFP (Golgi)	8.95 ± 3.47
DAPI (Nucleus)	6.81 ± 0.57

**Table 3:** Source and concentrations of the primary antibodies used in the study.

Name	Company	Class	Host	Antigen	Catalogue number	Dilution
Anti-ATPB	Abcam	Monoclonal	Mouse	Whole heart mitochondria (human)	ab14730	1:250 (IF)
Anti-MFN2	Abcam	Monoclonal	Mouse	a.a. 661–758 of MFN2	ab56889	1:5000 (WB)
Anti-GFP	Life Technologies	Polyclonal	Rabbit	GFP isolated from *Aequorea victoria*	A6455	1:400 (IF)
Anti-ERp72 (PDI)	Enzo Life Sciences	Polyclonal	Rabbit	a.a. 623–638 of ERp72	ADI-SPS-720	1:100 (IF)
Anti- α-Tubulin	EMD Millipore	Monoclonal	Mouse	a.a. 426–450 of α-Tubulin	05–829	1:50,000 (WB)
Anti-EEA1	Cell Signaling Technology	Polyclonal	Rabbit	Synthetic peptide corresponding to residues surrounding Gly14 of human EEA1	2411 S	1:100 (IF)

**Figure 4:**
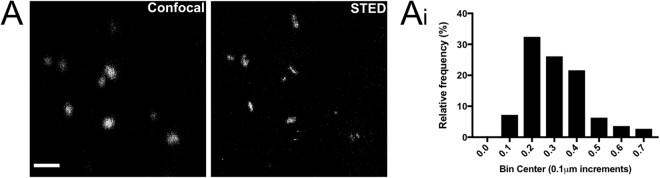
Super-resolution imaging of fluorescent BiFC puncta in NSC34 cells. Representative confocal image versus STED super-resolution image of the same region of an NSC34 cell transiently expressing the V1-ER and V2-Mito reporters (**A**). Scale bar, 1 μm. Pixel size = 0.2 μm. Frequency distribution graph illustrating the relative distribution of BiFC punctum size as measured by the straight line tool in ImageJ across the widest point of an individual fluorescent punctum (**B**). n = 111 BiFC puncta (7 cells total, mean value = 0.31 μm).

Many of the BiFC signals were at the limit of resolution for conventional confocal microscopy. In order to gain a more accurate measurement super resolution STED microscopy was done on NSC34 cells transiently expressing V1-ER and V2-Mito (Fig. 4). The pattern of signals was heterogeneous, with areas of association detected in a single optical section ranging in length between 100–700 nm. This super resolution microscopic analysis indicated that puncta are discrete single structures, the number of which may be quantified by conventional confocal microscopy.

**Figure 5:**
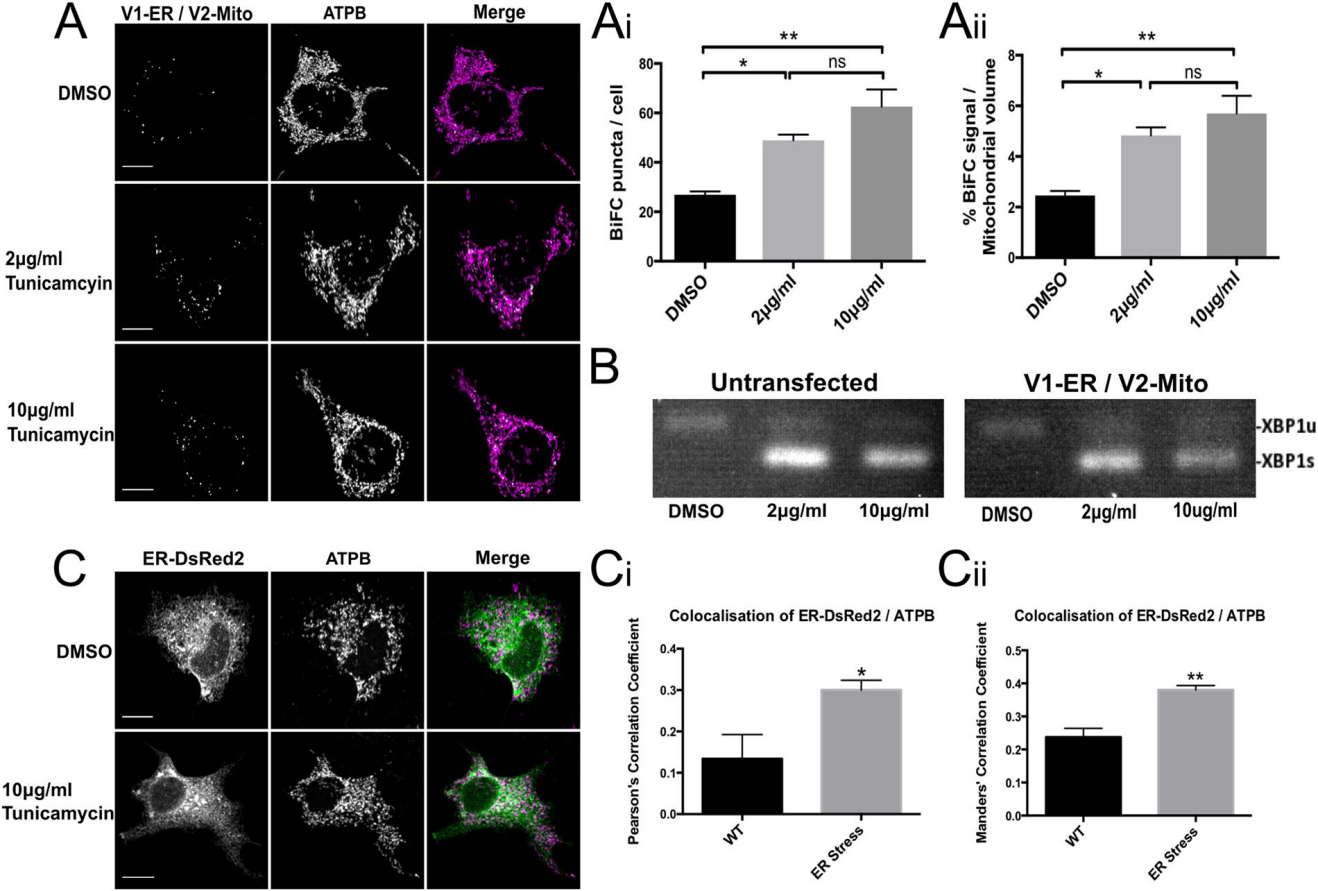
ER Stress significantly increases the number of ER-mitochondria contacts in NSC34 cells. NSC34 cells expressing V1-ER/V2-Mito were treated with 2 μg/ml or 10 μg/ml tunicamycin to induce an ER stress (**A**). ER stress significantly increases the mean number of BiFC puncta per cell in and (**A(i)**) significantly increases the total volume of BiFC signal as a percentage of total mitochondrial volume (**A(ii)**). n = 3 independent experiments (32–34 cells total). Values indicate mean values of three independent experiments. Error bars indicate SEM. Statistical significance was measured using one-way ANOVA followed by Holm-Sidak’s multiple comparison test, ns (non-significant) P > 0.05, *P < 0.05, **P < 0.01. ER stress was confirmed by detection of spliced XBP1 mRNA. Expression of the Venus reporter plasmids alone did not induce an ER stress (**B**). For comparison, in parallel NSC34 cultures the ER and mitochondria were labeled by co-expression of ER-DsRed2 (Green) and anti-ATPB (Magenta) respectively (**C**). A significant increase in the co-localisation of ER-DsRed and ATPB signals was detected following ER stress induced with 10 μg/ml tunicamycin as quantified by both Pearsons’ Correlation Coefficient (**C(i)**) and Manders’ Correlation Coefficient (**C(ii)**). n = 4 independent experiments (26 cells total). Statistical significance was measured using unpaired two-tailed t-test. *P < 0.05, **P < 0.01. Error bars indicate SEM.

ER stress has been shown to increase the amount of ER-mitochondria membrane association^34^. To test if the BiFC system was sensitive to this dynamic change an ER stress was induced in NSC34 cells co-expressing V1-ER and V2-Mito by incubating with 2 or 10 μg/ml tunicamycin for 6 hours. A robust ER stress response was induced at both drug concentrations as indicated by splicing of XBP1 mRNA^35^ (Fig. 5). The highest tunicamycin level induced a two-fold increase in the average number of punctate fluorescent signals per cell and an increase of similar magnitude in the size of this fluorescent signal as a percentage of the total mitochondrial volume, consistent with an increase in membrane associations (Fig. 5(Ai&ii)). Previous studies have used co-localisation analysis of fluorescent signals separately targeted to the ER and mitochondria as a measure of association between the membranes of the two organelles. To compare such co-localisation analysis with the BiFC method, ER stress was induced in parallel cultures and analysed by co-localisation of ER-targeted DsRed2 and the mitochondrial protein ATPB. In this case, co-localisation detected a similar increase in ER – mitochondria associations as that reported by the BiFC. These results are consistent with previous observations (Fig. 5C)^34^.

**Figure 6:**
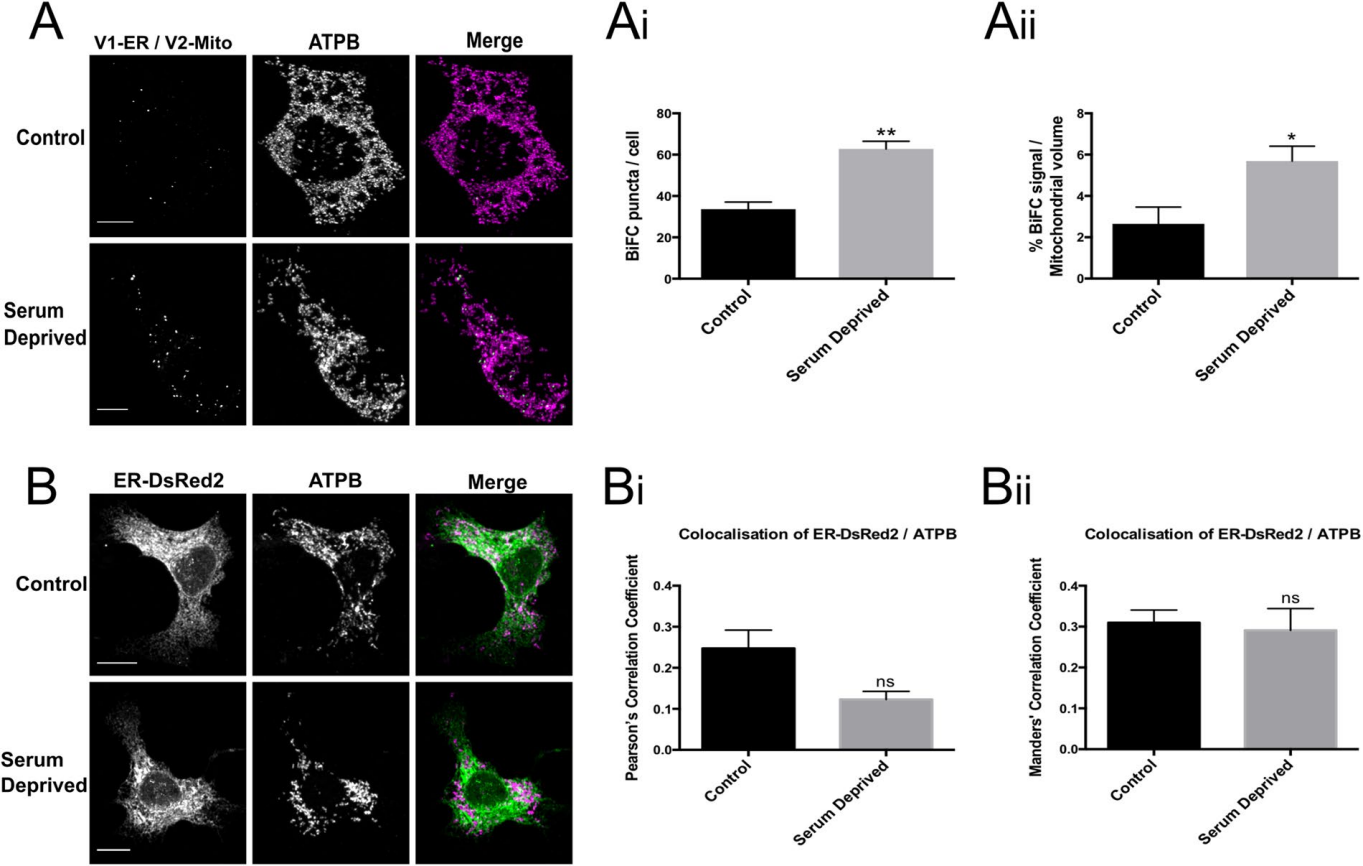
Serum deprivation significantly increases the number of ER-mitochondria contacts in NSC34 cells. NSC34 cells transiently expressing V1-ER/V2-Mito were grown in 10% FBS (control) or 0% FBS (serum deprived) for 24 h post transfection **(A)**. Serum deprivation significantly increases the mean number of BiFC puncta per cell (**A(i)**) and the total volume of BiFC signal as a percentage of total mitochondrial volume (**A(ii)**). n = 3 independent experiments (36–37 cells total). Statistical significance was measured using unpaired two-tailed t-test, *P < 0.05, **P < 0.01. Values indicate mean value of three independent experiments. Error bars indicate SEM. Co-localisation of ER and mitochondrial fluorescence does not detect a significant change in ER/mitochondrial contact **(B)**. In parallel NSC34 cultures the ER and mitochondria were labeled by co-expression of ER-DsRed2 (Green) and anti-ATPB (Magenta) respectively. No significant change in the co-localisation of ER and mitochondria was detected as measured by either Pearson’s Correlation Coefficient (PCC) (**B(i)**), or Mander’s Correlation Coefficient (MCC) (**B(ii)**). n = 3 independent experiments (19 cells total); ns (non-significant) P > 0.05. Error bars indicate SEM. Scale bars, 10 μm.

Serum deprivation has also been shown to increase the number of tight associations (<16 nm) between the ER and OMM^36^. 24 hrs following removal of serum, a significant increase in the number of fluorescent puncta was detected in NSC34 cells co-expressing V1-ER and V2-Mito (Fig. 6). However, in this case, co-localisation analysis of fluorescence from separately labelled ER and mitochondria did not detect a significant change as quantified by two different methods of analysis (Fig. 6). This illustrates that this BiFC method can detect changes in ER-OMM associations that may be undetected by simple co-localization analysis.

**Figure 7:**
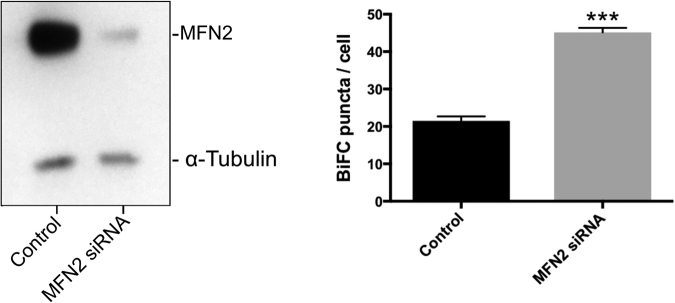
Mitofusin-2 (MFN2) levels influence the number of V1-ER/V2-Mito BiFC signals. siRNA was used to induced a knock-down of MFN2 to less than 20% of normal levels in NSC34 cells stably expressing V1-ER/V2-Mito (**A(i)**). This was associated with an increase the mean number of BiFC puncta per cell (**A(ii)**). n = 3 independent experiments (30–32 cells total). Values indicate the mean values from three independent experiments. Error bars represent SEM. Statistical significance was determined using unpaired two-tailed t-test, ***P < 0.01.

The role of Mitofusin-2 (MFN2) in maintaining ER/mitochondria associations has been controversial. Initial reports that reduced levels or absence of MFN2 resulted in less ER-mitochondrial contacts led to the suggestion that the protein acts as a tether between the two membranes^37^. However, more recent analysis of cells from *mfn2* (−/−) animals detected an increased level of ER-mitochondrial juxtapostion and Ca^2+^ coupling between the organelles^38,39^. Changes in mitochondrial shape may interfere with fluorescence-based co-localization reporter systems^37^ but the exact role of MFN2 in maintaining ER-mitochondria contacts remains controversial^40–42^. To further test the utility of the BiFC system, MFN2 levels were reduced in NSC34 cells using siRNA knockdown. An 80% reduction in MFN2 resulted in a significant increase in the number of fluorescent puncta detected by V1-ER/V2-Mito BiFC (Fig. 7). This suggests that the ER-OMM associations detected here by BiFC are not dependent on MFN2.

These results indicate that the BiFC system presented can report changes in ER-mitochondrial associations under three different conditions, and that in some instances it is more sensitive than previous fluorescence co-localization approaches.

## Discussion

A BiFC reporter system for close associations between the ER and mitochondria has been produced by targeting the two complementing fragments of the split Venus fluorescent protein to the cytoplasmic faces of the ER and the outer membrane of mitochondria. Tethering the complementing YFP Venus fragments with fusion proteins of different size generated distinct fluorescent signals, indicating that associations of different distance may be reported by a BiFC system. BiFC, therefore, may be used to distinguish between membrane associations of varying distance, which are thought to have specific functional roles^36,43^. The closest contacts as detected by the V1-ER/V2-Mito combination formed on a subset of mitochondria and were heterogeneous in size ranging from 100–700 nm in length. Csordas *et al*.^44^, artificially forced the close association of ER and OMM by expressing a fluorescent fusion protein containing both an ER targeting sequence and a mitochondrial targeting sequence. In that case, the fusion protein had 84 amino acids in addition to the fluorescent protein, and resulted in an association between the ER and OMM with a distance of approximately 6 nm between the two membranes^36^. The combination of the V1-ER and V2-Mito fusion proteins adds a total of 85 additional amino acids to the Venus protein, suggesting that the contact sites reported by the V1-ER/V2-Mito combination are of similar dimensions to those previously reported^36^. Assuming the extra-membrane residues to be in a fully extended conformation would give an upper estimate of the largest gap compatible with fluorescent protein formation of approximately 10 nm. Therefore the distance between the ER and mitochondrial membranes detected by this BiFC combination is most likely in the range of 6 to 10 nm.

Large receptor proteins and complexes such as the IP_3_R and ribosomes may impose limits on the close association of the ER and OMM. Giacomello and Pellegrini have proposed that different gaps between the membranes reflect functionally distinct domains participating in Ca^2+^ signalling, lipid biosynthesis and transfer, and autophagocytosis^43^. The predicted 6–10 nm gap identified here would be consistent with sites of lipid transfer mediated by members of the tubular lipid-binding (TULIP) superfamily of membrane proteins, forming a 9 nm long channel that may mediate lipid transfer between organelles (reviewed^45^). Increased associations between ER cisternae and mitochondria are made following induction of the Unfolded Protein Response^46^. Since ribosomes are localised to ER cisternae the observation that BiFC puncta numbers increase following tunicamycin treatment suggests that V1-ER and V2-Mito can interact at ER cisternae despite the restrictions imposed by ribosome size.

When tight contacts between the ER and OMM are induced artificially, gross changes in organelle morphology can occur over time^44^. In contrast, the pattern of the V1-ER/V2-Mito BiFC signal is generally stable over time with no gross changes in ER or mitochondrial morphology seen in transient or stably transfected cells. Therefore the association of the separate halves of the Venus protein appears insufficient to disrupt the normal pattern of ER-mitochondria associations. The BiFC system complements existing methods to detect organelle associations. Electron microscopy has the highest resolution however localising specific proteins to particular regions of apposition requires correlative electron and fluorescent microscopy or immune gold labelling, both of which are technically very challenging and must be done on fixed material. Fluorescent proximity ligation assays are more readily quantified and can detect specific proteins at regions of contact (25). Again, however, this method is used on fixed samples. Fluorescent protein- based methods have the significant advantage that they can be readily detected in living cells or tissues. An example of BiFC in living cells in shown in Supplementary video S4. Co-localization of distinct fluorescent proteins localised on separate membranes has a limited resolution and cannot distinguish between contacts less than approximately 150 nm. Dimerization dependent Fluorescent proteins (ddFPs) and BiFC have improved detection limits as they both depend on the direct association of polypeptides targeted to separate membranes. In both cases the proximity of membrane associations that can be detected depends on the size of the targeting and linker sequences of the respective fusion proteins. One potential advantage of a BiFC approach over ddFPs is a reduced background as the complementing halves of the YFP show no fluorescence. The resulting improvement of signal to noise would allow the detection of weaker transient signals.

The average number of BiFC puncta increases in response to homeostatic stress and reduction in MFN2 levels. This seems incompatible with the proposal that MFN2 is required to maintain close ER-mitochondria associations. However, it is possible that the V1-ER/V2-Mito BiFC as configured may detect MFN2-independent contacts induced following a mitochondrial stress resulting from a reduction of MFN2 levels. Alternatively, MFN2 may not be directly responsible for maintaining ER-mitochondria associations as suggested. The magnitude of the increase in V1-ER/V2-Mito signal following MFN2 knock-down here is similar to that seen for ER-mitochondria fluorescence co-localisation in studies using *mfn2* ^−/−^ mice^38^.

All mitochondria appear to come into contact with the ER, as indicated by the V1-VAPB/V2-Mito BiFC signal (Fig. 2A) and previously using dimerization-dependent fluorescent proteins^27^. However, not all mitochondria have an ER association detectable by V1-ER/V2-Mito. Ultrastructure analysis of mouse liver indicated that 25% of mitochondria have close ER associations and it is suggested that this heterogeneity is a cell type specific characteristic and changes in response to the physiological environment^23^. The mitochondria identified as interacting with the ER by this BiFC system might therefore have distinct morphologies, respiratory rates and/or, distinct Ca^2+^ signalling activities.

Subtle changes in distances between ER and mitochondrial membranes are not measurable by co-localization of fluorescence as they may fall bellow the resolution limit of conventional optical microscopy. Notably, induction of an Unfolded Protein Response produced changes in ER mitochondria associations that were detectable by both the V1-ER/V2-Mito BiFC and fluorescence co-localisation. In contrast, serum removal only produced a significant change in V1-ER/V2-Mito BiFC puncta number, and not fluorescence co-localisation. BiFC, therefore, may represent a more sensitive *in vivo* technique that also avoids the technically challenging ultrastructure analysis using electron microscopy on fixed samples.

The increase in BiFC puncta following tunicamycin treatment is consistent with studies showing increases in ER mitochondrial contacts in the early phases of ER stress^24^. It may also be the case that the associations detected by BiFC are related to sites of mitochondrial fusion and biogenesis^34^, and the signal is somewhat reminiscent of Miro1 labelling in COS-7 cells^47^. However, it has been suggested that mitochondria biogenesis is independent of UPR stress activity from the ER^48^. Further studies are required to determine if these mitochondria contacted via these BiFC puncta have particular properties relating to biogenesis, Ca^2+^ signalling or respiratory activity.

## Materials and Methods

### Plasmids and primers

V1-VAPB has been described previously^49^. V1-ER was derived from V1-VAPB by a deletion PCR mutagenesis using the primers gggtcctccggaatgggcctgagcgcccggctgct and cattccggaggacccaccacctccagagc generating a Venus 1–C terminal fusion with the C-terminal 25 amino acids from mouse VAPB, GLSARLLALVVLFFIVGVIIGKIAL.

V2-Mito contains the first 34 amino acids of human TOMM20, fused to the terminus of Venus(2). The coding sequence for MVGRNSAIAAGVCGALFIGYCIYFDRKRRSDPN was generated from the human TOMM20 cDNA by a PCR using oligonucleotide primers cggcggccgctccggaccatggtgggccggaacagcgcc and atctagattaatcgattgcagacccgatgccgctgc, The PCR product was digested and ligated into the NotI and ClaI restrictions sites of pcDNA3.1 Venus(2)-zip. (Generous gift of S.W.Michnick), to create Mito-V2 fusion gene.

Primers used for RT-PCT across slice junction of mouse XBP1 mRNA: ccaaggggaatgaagtgagg and aagggaggctggtaaggaac.

### Cell culture and transfection

NSC34^50^, COS-7^51^, and HEK-293^52^ cells were maintained at 37 °C and 5% CO_2_ in Dulbecco’s modified high glucose Eagle’s medium supplemented with 10% fetal bovine serum and 1% antibiotic-antimycotic (Thermo Fisher Scientific). For imaging, cells were plated on glass coverslips coated with poly-L-lysine (50 μg/ml) and bovine plasma fibronectin (10 μg/ml) (Life Technologies). Plasmid transfections were done using Lipofectamine 2000 (Invitrogen) in Opti-MEM media (Thermo Fisher Scientific). Cells were imaged or harvested 48 h post-transfection. V1-ER and V2-Mito reporter plasmids were transfected at a 1:1 ratio at a total DNA of concentration of 1 μg per well of a 12 well dish (except where otherwise indicated). MFN2 siRNA (Dharmacon) transfection was performed with lipofectamine RNAiMAX at an siRNA concentration of 50 nM for 48 h.

Stably transfected NSC34 cells co-expressing V1-ER and V2-Mito were selected on 400 μg/ml zeocin (InvivoGen).

ER stress was induced with tunicamycin (Calbiochem) at 2 μg/ml or 10 μg/ml for 6 h, with an equivalent volume of DMSO as vehicle only control.

### Immunoblot analysis

Cell proteins were extracted in RIPA buffer (1.0% NP40, 0.1% SDS (w/v), 150 mM NaCl, 0.5% (w/v) Sodium deoxycholate, 50 mM Tris pH 8.0, Complete protease inhibitors (Roche)), and quantified by BCA protein assay (Thermo Scientific). Samples in SDS sample buffer (80 mM Tris HCl pH6.9, 2%(w/v) SDS, 100 mM DTT, 10% (v/v) glycerol, were heated at 80 °C for 15 min, prior to separation on NuPAGE 4–12% Bis-Tris Gel (Invitrogen) in MES SDS running buffer (Invitrogen). Proteins were then transferred to PVDF Immobilon P (EMD Millipore) in NuPAGE transfer buffer contain 10% (v/v) methanol. Non-specific protein binding sights on the membranes were blocked by incubating for at least 60 mins in PBS containing 5% (w/v) non-fat dry milk and 0.1% (v/v) Tween-20. Primary antibodies were used in blocking solution at indicated concentrations overnight at 4 °C, following which membranes were washed at room temperature 3 times for 5 min in PBS containing 0.1% (v/v) Tween-20. Bound antibody was detected using horseradish peroxidase-conjugated secondary antibodies at 1:5,000 dilution (Jackson ImmunoResearch) followed by ECL detection (Amersham GE Life Sciences) using X-ray film (FUJIFILM). Band intensities were quantified by densitometric analysis using ImageJ.

### Immunocytochemistry

Cells were fixed in 4% (w/v) paraformaldehyde in phosphate buffered saline (PBS) for 15 min at room temperature, except for anti-ERp72 where methanol at −20 °C was used for 5 min. After washing with PBS, cells were permeabilised with 0.4% (v/v) Triton X-100 for 10 min at room temperature. Cells were then incubated in a blocking solution consisting of PBS, 0.2% fish gelatin and 0.02% saponin (PGAS), at 4 °C overnight. Primary and secondary antibodies were diluted in PGAS. Primary antibodies were incubated at room temperature for 30–60 min. Following each incubation cells were washed 3 times with PGAS and an additional final 3 washes were carried out using PBS before coverslips were mounted using Vectashield with DAPI (Vector Laboratories, Inc. Burlingame, CA 94010) or ProLong Diamond Antifade mountant (Thermo Fisher Scientific).

Secondary antibodies used were from Jackson ImmunoResearch. Horseradish-peroxidase conjugated goat anti-rabbit (111-035-003), donkey anti-mouse Cy2 (715-225-150), Cy3 and Cy5-coupled goat anti-mouse (115-165-146, 115-175-156), Cy3 and Cy5-coupled donkey anti-Rabbit (711-175-152, 711-175-152).

### RNA extraction and PCR

Total RNA was extracted from NSC34 cells using the RNeasy mini kit (Qiagen) as per manufacturers instructions. cDNA was generated using the Omniscript reverse transcription kit (Qiagen) followed by PCR amplification using Taq DNA polymerase (Qiagen). PCR products were visualised on a 3.5% (w/v) TAE agarose gel stained with SyberSafe (Invirogen).

### Confocal Microscopy

Confocal images were acquired using a Nikon A1R microscope. For the quantification and colocalisation analysis experiments, z-stack images were taken with a pixel size of 60 × 60 nm^2^ and z-step size of 150 nm using a 60X (Plan ApoVC NA 1.4 oil) objective. Excitation laser wavelengths were for YFP (Venus) 488 nm, Cy3 and DsRed 561 nm, CFP and DAPI 401.5 nm and Cy5 641 nm. For comparison of the relative levels of protein expression shown in SFig. 2, single plane images were acquired with a 20X objective lens (Plan Apo NA 0.75) with excitation laser wavelengths set at 401.5 nm (DAPI) and 561 nm (Cy3) by keeping the same laser power, detector gain and acquisition parameters. Microscope control and image acquisition were done using the NIS-Elements-v4.13 software.

### Live-Cell Imaging

NSC34 cells were plated on poly-D-lysine/fibronectin coated 35 mm glass-bottomed petri dishes (14 mm microwell diameter, glass thickness No. 1.5) (MatTEK). Mitochondria were labeled with 25 nM MitoTracker CMXRos (Invitrogen) for 30 min in pre-warmed DMEM, followed by three five minute washes with DMEM. Two channel, single plane imaging of live-cells was performed in a heated chamber at 37 °C with 5% CO_2_ (Okolab) using a 60X objective lens (Plan ApoVC NA 1.4 oil) at an acquisition rate of 0.25 fps.

### CW-gSTED microscopy

Continuous wave gated stimulated emission depletion (CW-gSTED) microscopy was done using a Leica SP5 confocal laser scanning microscope equipped with a 592 nm depletion laser and 100 × 1.4 NA HCX PL Apo oil immersion objective lens. YFP was excited using a white light laser tuned to 514 nm with an 80 MHz pulse rate. STED was achieved by concurrent 592 nm depletion aligned to the excitation laser. 524–580 nm emission was isolated by an acousto-optical beamsplitter and detected with a Leica HyD hybrid detector gated to detect 0.5–8 ns following each excitation pulse. Single slice images were acquired with a 0.02 µm pixel size to achieve Nyquist sampling.

### Image analysis

Deconvolution of confocal images was performed using Huygens Essential (Huygens Software 4.5.1p3) before subsequent analysis. Colocalisation analyses were done on 3D, deconvolved images and quantified using ImarisColoc (Imaris v8.2.1, Bitplane Inc, software available at http://bitplane.com). Quantification of BiFC puncta was automated using the 3D volume rendering function of Imaris followed by the ‘total number of disconnected components’ measurement. For quantification of the signal overlap between fluorescent puncta and organelle markers (Fig. 3) first the number of BiFC puncta in a cell was quantified on Imaris with the ‘Spots’ function using the maximum intensity value as a threshold. Using this same threshold intensity value, a colocalisation channel was built between the BiFC and organelle marker channel. Finally, the number of spots in the colocalisation channel was quantified in the same manner and expressed as a percentage of total BiFC puncta. Analysis of images acquired by STED microscopy was carried out using the “straight line” tool in ImageJ. A straight line was drawn manually across the point of widest distance of each individual fluorescent puncta and the length of the line calculated in μm.

All image analysis and quantification of images acquired with confocal microscopy were performed on deconvolved images without any additional processing. Brightness and contrast settings were adjusted in ImageJ for presentation purposes only.

### Statistical analysis

All experiments were repeated at least three times for statistical analysis. Statistical analysis was carried out using Prism version 6.0 h (GraphPad). Unless otherwise stated, statistical significance was measured using unpaired two-tailed t-test or one-way ANOVA followed by Holm-Sidak’s multiple comparison test; *P < 0.05, **P < 0.01, ***P < 0.001.

## Electronic supplementary material

Supplementary figures

Supplementary Figure S4
