# Supplementary material for: A Bi-fluorescence complementation system to detect associations between the Endoplasmic reticulum and mitochondria
Source: Sci Rep. 2017 Dec 12;7:17467. doi: 10.1038/s41598-017-17278-1 (PMC5727038; doi:10.1038/s41598-017-17278-1)

Title:

A Bi-fluorescence complementation system to detect associations between the Endoplasmic reticulum and mitochondria.

Mr. Mark Harmon*

Dr. Philip Larkman

Prof. Giles E. Hardingham

Dr. Mandy Jackson

Dr. Paul Skehel**

Centre for Integrative Physiology,

Euan MacDonald Centre for Research into motor neuron disease.

The University of Edinburgh.

* Supported by an International Career Development PhD Scholarship funded through the Zhejiang University – University of Edinburgh Institute.

** Corresponding author.

Author contributions: MH: Data Curation, Formal Analysis, Investigation, Methodology, Original Visualization, Writing and Reviewing. **PL**: Supervision, Writing and Reviewing. **GH**: Supervision, Writing and Reviewing. **MJ**. Supervision, Resources, Conceptualization, Writing and Reviewing. **PS**:Conceptualization, Investigation, Methodology, Resources, Funding Acquisition, Supervision, Original Writing.

The authors declare no conflict of interest relating to the work reported.

Supplementary figure S1. Venus fusion proteins do not generate fluorescence unless co-expressed with the complementary venus fragment. V1-VAPB, V1-ER and V2-Mito were expressed in NSC34 cells either alone or with the complementary venus fusion protein. Immunofluorescent staining with an anti-GFP antiserum (magenta) recognising both venus fragments confirmed the expression of the fusion proteins. A YFP BiFC signal was only detected when venus 1 and venus 2 fusion proteins were co-expressed (green signal, bottom two rows).

Supplementary figure S2. Coexpression of V1-ER and V2-Mito generates an similar BiFC signal in multiple cell lines. Discrete puncta of fluorescence is generated when V1-ER and V2-Mito are transiently exoressed in three separate cells lines NSC34, COS-7 and HEK293. In all cases punta are associated with mitochondria labelled with anti-ATPB.

Supplementary figure S3. Increasing total DNA concentration of V1-ER and V2-Mito does not significantly alter the mean number of BiFC puncta per cell. (A) NSC34 cells transfected with V1-ER and V2-Mito expression plasmids in a 1:1 ratio at a total DNA concentration of 0.5μg, 1μg or 1.5μg were imaged by confocal microscopy and the mean number of BiFC puncta per cell was quantified on Imaris image analysis software (as detailed in the methods section). Statistical significance was determined by one-way Anova followed by Holm-Sidak's multiple comparison test. No significant change was detected between any of the experimental groups. Values indicate the mean number of BiFC puncta per cell. n = 3 independent experiments (23-27 cells total). Error bars represent SEM. **(B)** An equivalent increase in the levels of proteins produced with increasing total DNA concentration was determined by anti-GFP immunostaining (Red) of transfected and untransfected NSC34 cells. Nuclei were labelled with DAPI (Blue). Random areas of the coverslips were imaged on a confocal microscope with identical image acquisition settings (20x). An increase in protein production was illustrated by an increase in both the total number of transfected cells and an increase in brightness of the anti-GFP signal.

Supplementary video S4: Live-cell imaging of NSC34 cell expressing V1-ER and V2-Mito reporter plasmids. NSC34 cells were plated and transfected on poly-D-lysine/fibronectin coated glass-bottomed petri dishes (No. 1.5, MatTEK). Mitochondria were labeled using MitoTracker CMXRos (Invitrogen, 25nM for 30 min in pre-warmed DMEM, followed by three washes of 5 min each with DMEM). Cultures were maintained at 37°C / 5% CO_2, while two channel, single plane images were acquired using a 60X objective (Plan ApoVC NA 1.4 OIL) at a rate of 0.25 fps for 8 mins. Mitochondria (cyan), BiFC (green).

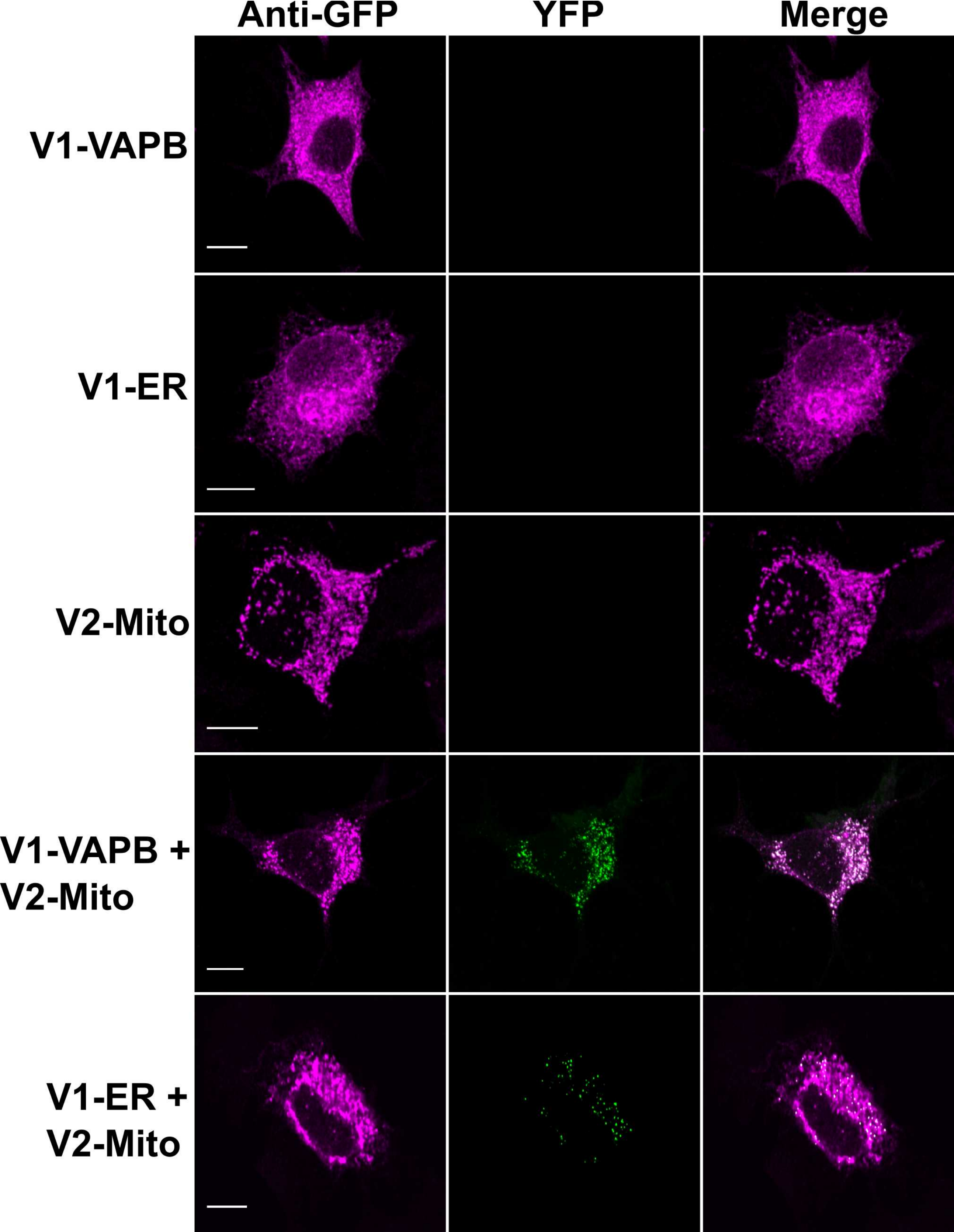
Anti-GFP
YFP
Merge
V1-VAPB
V1-ER
V2-Mito
V1-VAPB + V2-Mito
V1-ER + V2-Mito

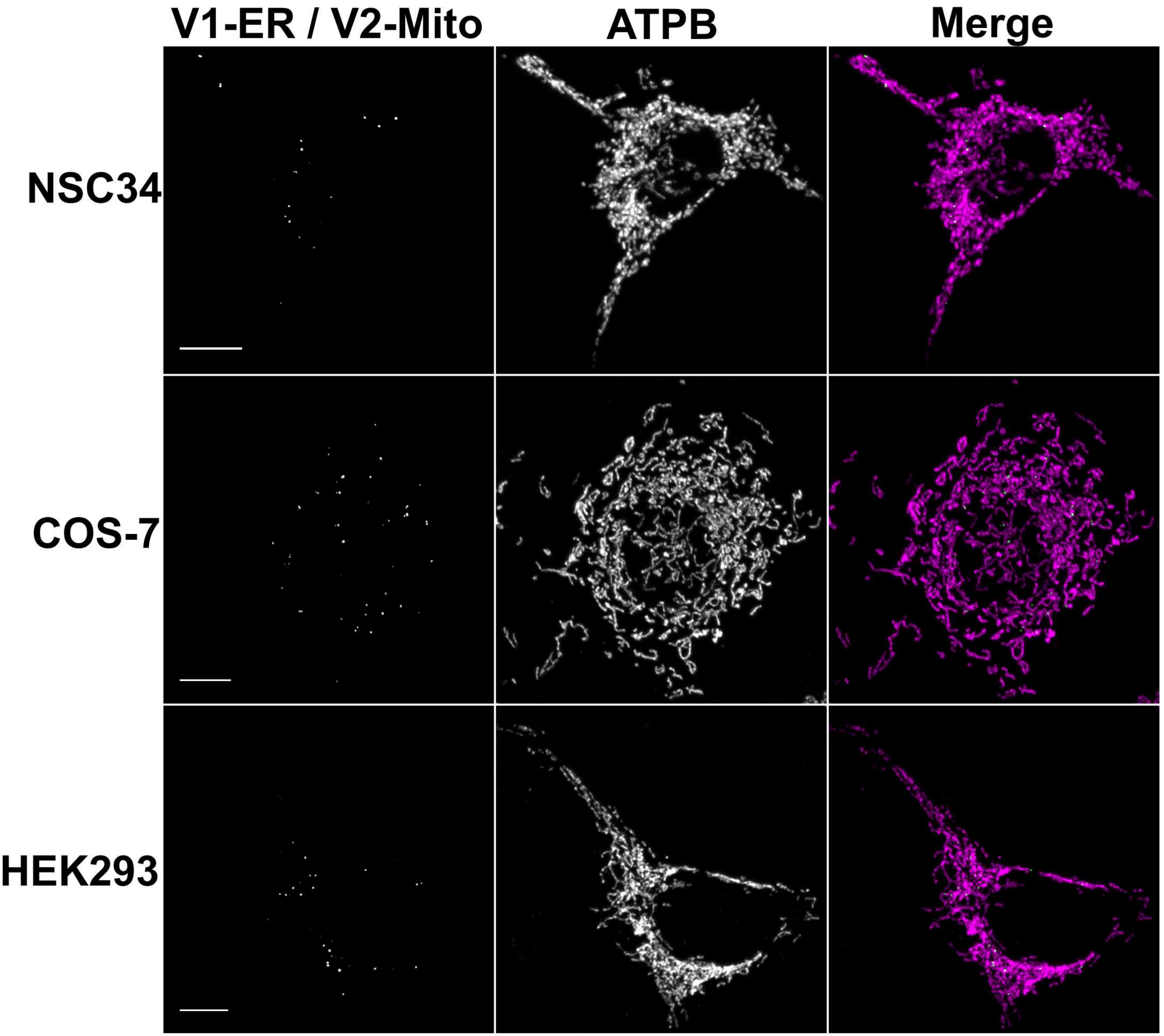
V1-ER / V2-Mito
ATPB
Merge
NSC34
COS-7
HEK293

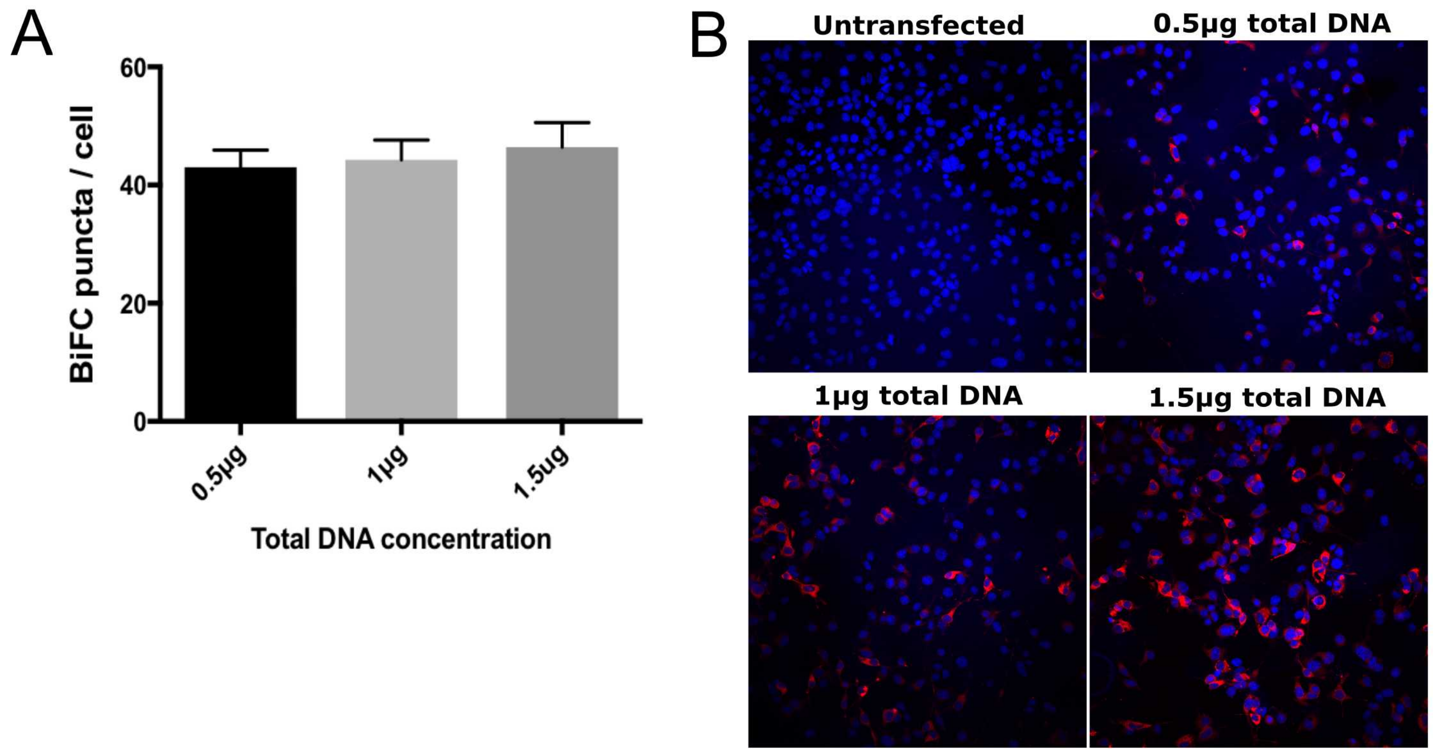

A
60
40
20
0
BiFC puncta / cell
0.5μg
1μg
1.5μg
Total DNA concentration
B
Untransfected
0.5μg total DNA
1μg total DNA
1.5μg total DNA

Full gel analysis of RT-PCR analysis of xbp1 mRNA splice from Fig 5.

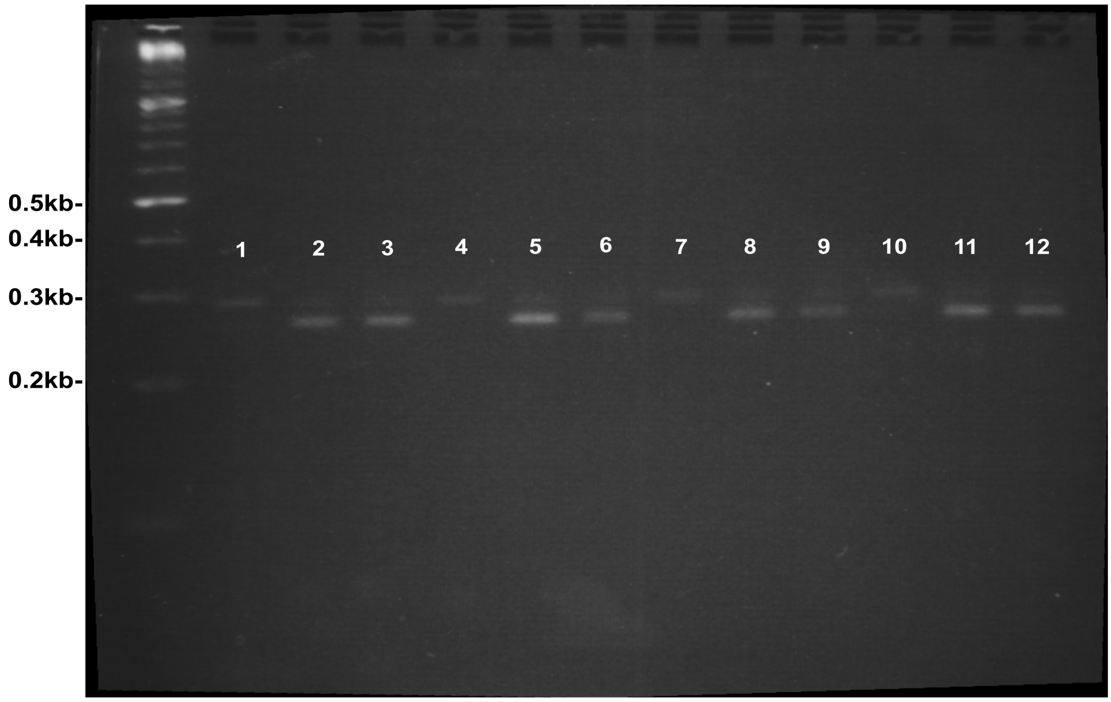

1. Untransfected - DMSO
2. Untransfected - 2µg Tunicamycin
3. Untransfected - 10µg Tunicamycin

4. V1-ER alone - DMSO
5. V1-ER alone - 2µg Tunicamycin
6. V1-ER alone - 10µg Tunicamycin

7. V2-Mito alone - DMSO
8. V2-Mito alone - 2µg Tunicamycin
9. V2-Mito alone - 10µg Tunicamycin

10. V1-ER/V2-Mito - DMSO
11. V1-ER/V2-Mito - 2µg Tunicamycin
12. V1-ER/V2-Mito - 10ug Tunicamycin

Full blot of Mitofusin-2 expression shown in Fig 7.

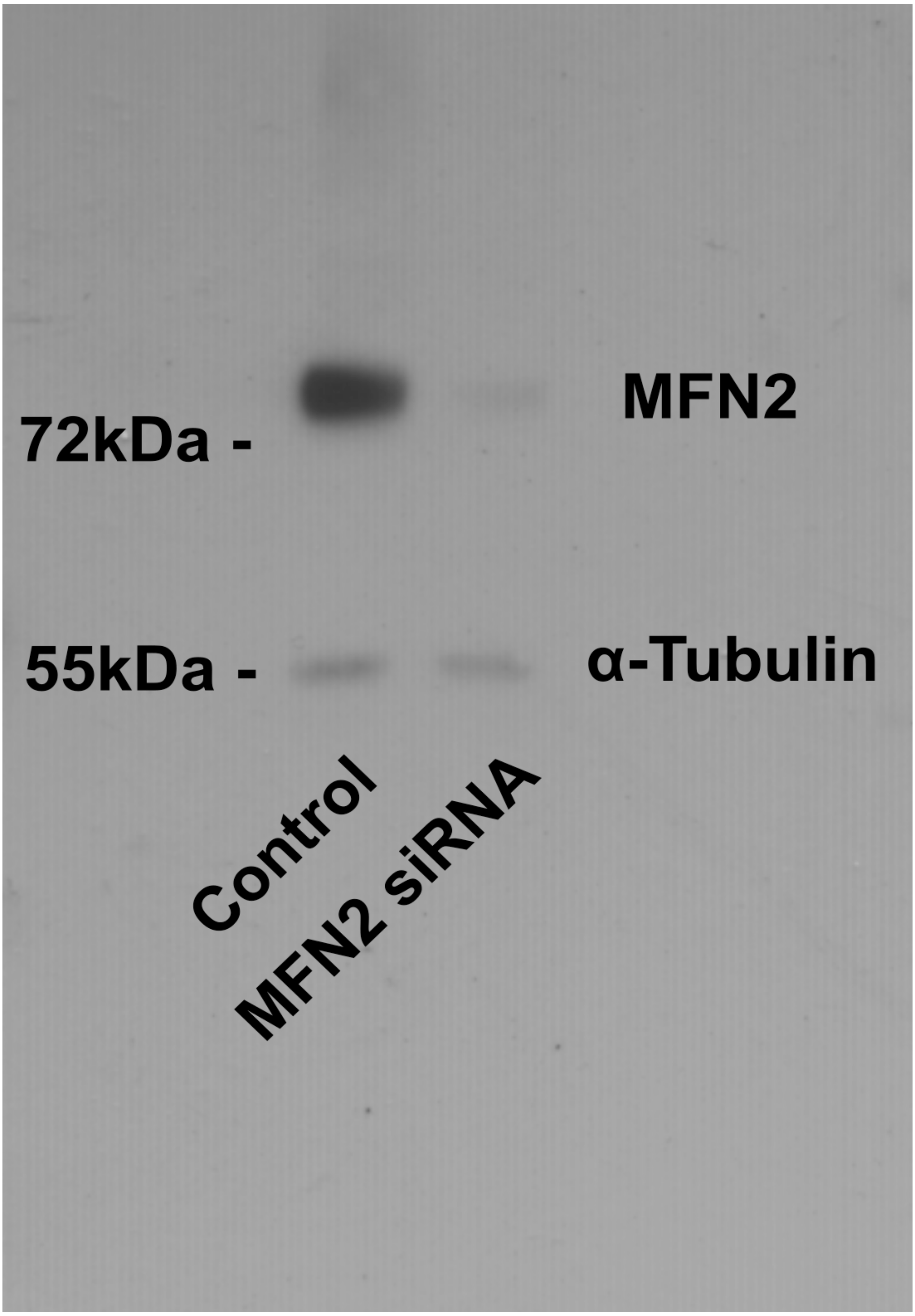

Supplement: Supplementary file 1 — Supplementary figures [file 41598_2017_17278_MOESM1_ESM.pdf]
